# Type 1 Diabetes and COVID-19: A Literature Review and Possible Management

**DOI:** 10.5812/ijem-139768

**Published:** 2023-10-23

**Authors:** Kebria Kashfi, Narges Anbardar, Artin Asadipooya, Kamyar Asadipooya

**Affiliations:** 1 Department of Clinical Medicine, Florida International University AUACOM, Florida, USA; 2 Department of Clinical Medicine, SMUSOM, Cleveland Clinic Lerner College of Medicine, Ohio, USA; 3 Department of Neuroscience, University of Kentucky, Lexington, Kentucky, USA; 4 Division of Endocrinology, Diabetes, and Metabolism, Department of Medicine, Barnstable Brown Diabetes and Obesity Center, University of Kentucky, Lexington, Kentucky, USA

**Keywords:** ACE2, DKA, SARS-CoV-2, T1D

## Abstract

**Context:**

Severe acute respiratory syndrome coronavirus 2 (SARS-CoV-2) infection normally damages the respiratory system but might likewise impair endocrine organs’ function. Thyroid dysfunction and hyperglycemia are common endocrine complications of SARS-CoV-2 infection. The onset of type 1 diabetes (T1D) and associated complications, including diabetic ketoacidosis (DKA), hospitalization, and death, are thought to have increased during the coronavirus disease 2019 (COVID-19) pandemic. The aim of this study was to review the available data about the incidence rate of T1D and accompanying complications since the beginning of the COVID-19 pandemic.

**Evidence Acquisition:**

A literature review was conducted using the electronic databases PubMed and Google Scholar. The keywords “T1D, T1DM, Type 1 DM or Type 1 Diabetes”, “Coronavirus, SARS-CoV-2 or COVID-19” were used to search these databases. Titles and abstracts were screened for selection, and then relevant studies were reviewed in full text.

**Results:**

A total of 25 manuscripts out of 304 identified studies were selected. There were 15 (60%) multicenter or nationwide studies. The data about the incidence rate of T1D, hospitalization, and death are not consistent across countries; however, DKA incidence and severity seem to be higher during the COVID-19 pandemic. The present study’s data collection demonstrated that COVID-19 might or might not increase the incidence of T1D. Nevertheless, it is associated with the higher incidence and severity of DKA in T1D patients. This finding might indicate that antivirals are not fully protective against the endocrine complications of SARS-CoV-2 infection, which promotes the application of an alternative approach.

**Conclusions:**

Combining medications that reduce SARS-CoV-2 entry into the cells and modulate the immune response to infection is an alternative practical approach to treating COVID-19.

## 1. Context

The coronavirus disease 2019 (COVID-19) pandemic, caused by severe acute respiratory syndrome coronavirus 2 (SARS-CoV-2), remains a prominent global health concern. Severe acute respiratory syndrome coronavirus 2 is a ribonucleic acid (RNA) virus belonging to the family Coronaviridae. There are different variants with unique mutations, which mainly affect the respiratory system but can also damage the nervous system and endocrine organs. The clinical manifestations of COVID-19 vary from asymptomatic or mild disease to acute respiratory distress syndrome (ARDS), hospitalization, and death (1). The commonly reported endocrine complications are thyroid dysfunction and hyperglycemia (2). The connection between COVID-19 and endocrine disorders, such as diabetes mellitus (DM), is a nuanced area of research.

Diabetes mellitus is a metabolic disorder characterized by a defect in insulin secretion, insulin function, or both, causing hyperglycemia and other debilitating complications, including micro- and macro-vascular complications. The two main classifications of DM, namely type 1 diabetes (T1D) and type 2 diabetes (T2D), differ in that T1D consists of reduced insulin production; nevertheless, T2D consists of inadequate cellular response to insulin signaling (3). Coronavirus disease 2019 can potentially increase the incidence rate of T1D and T2D. Type 1 diabetes typically begins with immune-mediated damage to pancreatic cells, which is triggered by genetic or environmental factors. Viral infections, such as enteroviruses and respiratory viruses, are probably responsible for autoimmunity against β-cells (4, 5). Generally, COVID-19 and T2D appear to have a bidirectional relationship; however, the relationship between COVID-19 and T1D remains controversial and multi-faceted (6).

Diabetic ketoacidosis (DKA) is a life-threatening complication that usually occurs in patients with T1D. In T1D, the absence of insulin promotes an excess breakdown of fats as an alternative source of energy, resulting in the buildup of acidic ketones and disrupting organ functions. During the COVID-19 pandemic, there was a notable increase in the incidence and severity of DKA in patients with T1D, suggesting a possibility that COVID-19 and DKA are causally connected (7-9). However, other studies found no evidence of a physiological association between DKA and SARS-CoV-2, implying that the surge in the incidence and severity of DKA during COVID-19 is best attributed to a diminished quality of care for diabetic patients due to an overburdened healthcare system (10-12). Moreover, although the severity of symptoms of T1D might have been exacerbated during the pandemic (once again, arguably due to strains on the healthcare system), the overall incidence rate of T1D might not have necessarily been impacted (13). Therefore, the interplay between COVID-19 and T1D is rather complex.

Although several systematic reviews have reported that SARS-CoV-2 infection can increase the risk of new-onset type 1 diabetes (NT1D) (14-17), no alternative approach was proposed to reduce the endocrine complications of COVID-19. This review aims to elucidate the intricate relationship between COVID-19 and T1D, emphasizing the different aspects of epidemiology, complications, and possible therapeutic strategies to improve their outcomes and mitigate mortality and morbidity associated with both diseases.

## 2. Evidence Acquisition

### 2.1. Data Sources and Searches

According to PRISMA guidelines (18), a systematic search was conducted in PubMed and Google Scholar for relevant studies. Search dates were within January 2020 and May 2023. The following keywords were applied for search: “T1D, T1DM, Type 1 DM or Type 1 Diabetes, Coronavirus, SARS-CoV-2 or COVID-19”.

### 2.2. Study Selection

**Figure 1.**
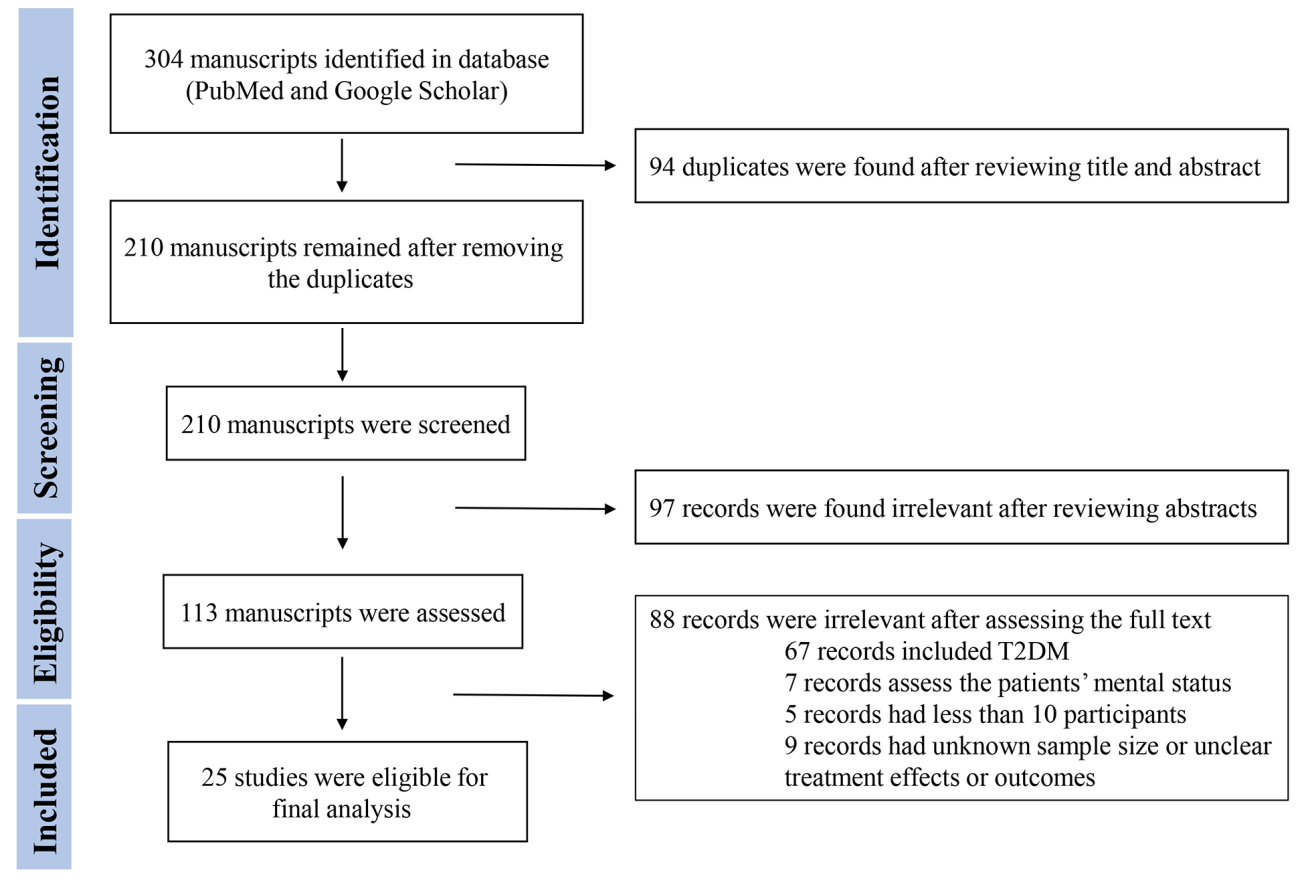
Flow diagram of the screening process of literature review (PRISMA flow diagram)

Two authors (KK and NA) reviewed abstracts, and a third author (KA) made a cross-check. The references of relevant reviews were reviewed to include further potentially relevant articles. Two authors contributed independently to the selection process, data extraction, and data collection. The participants, study type, outcomes, and interventions were used to select the relevant studies. The selected studies were discussed to resolve disagreements, and a third author participated if needed. This study reviewed (1) clinical research articles, such as cohorts, cross-sectional studies, case-control studies, and case series; (2) review articles, including mini-reviews, systematic reviews, and meta-analyses; and (3) opinion and commentary articles, such as editorials, commentaries, perspectives, and letter to editors, that discuss the incidence, clinical characteristics, outcomes, complications, morbidity, and mortality of T1D in COVID-19 patients or vice versa. This study included multicenter, nationwide, or observational original studies that were cohort, cross-sectional, or case-control and reported the complications or incidence of NT1D during the COVID-19 pandemic. This study also reviewed the data from two systematic reviews by Nassar et al. (14) and D'Souza et al. (17). Three reviewers (KK, NA, and KA) evaluated the risk of bias in the selected studies to make sure cohort, cross-sectional, or case-control studies were included. Figure 1 shows the flowchart for the systematic review.

Duplicates were eliminated after a review of all recognized articles from the initial searches. The remaining papers were read in full. The publications were summarized in terms of the author, journal, year of publication, country of origin, study design, number of participants, type of intervention, age, gender, outcomes (e.g., death, DKA, or other complications), new-onset diabetes or worsening of pre-existing diabetes, and other results in general. Most studies were observational or nonrandomized studies. This study assessed the risk of bias and quality by producing review-specific questions and guidance, constructing a flow diagram for the study, and judging bias and applicability. A formal narrative data synthesis was performed to investigate the effects of COVID-19 infection on the incidence of T1D and associated complications (e.g., DKA and death). The consistencies or discrepancies among the studies were discussed.

## 3. Results

A total of 304 studies were found during the initial database searches. The exclusion process resulted in 25 eligible manuscripts for further investigation (7-13, 19-36). Tables 1 and 2 show summaries of the baseline characteristics of the included studies.

**Table 1.** Outcomes and Population of Included Studies with Type 1 Diabetes (T1D) and Coronavirus Disease 2019 (COVID-19) Listing the Countries in Alphabetical Order

Author	Study Design and Population	Sample Size	Age	Gender (Male), No. (%)	Death, DKA, or NT1D	Comments
**Birkebaek et al. (** 7 **)**	An international multicenter study from 13 national diabetes registries, children and adolescents diagnosed with T1D (104290 children and adolescents)	8209 in 2020	9.9	4521 (55)	39.4% in 2020 (DKA at T1D diagnosis)	There was an exacerbation of DKA prevalence in T1D patients during the COVID-19 pandemic.
		8853 in 2021	9.5	4941 (55.8)	38.9% in 2021 (DKA at T1D diagnosis)	
		87228 within 2006-2019	9.5	47066 (54)	27.3% (23775) DKA at T1D diagnosis	
**Lawrence et al. (** 8 **)**	Retrospective cohort study, children < 18 years with the initial diagnosis of T1D	11 (Mar-May 2020)	8	27	73% DKA; 45% severe DKA; 11 NT1D	A significant increase in the severe DKA at the presentation of NT1D during the COVID-19
		42 (Mar-May 2015-2019)	7.9-10.2	33-63	26% DKA; 5% severe DKA; 9 (6-10) NT1D	
**Li et al. (** 9 **)**	Retrospective cohort study, hospitalized patients with COVID-19	658	57.3	297 (45.14)	64 (9.7%) death; 3 (0.005%) DKA	COVID-19 infection caused ketosis or ketoacidosis. COVID-19 induced DKA in diabetic patients. Ketosis increased the length of hospital stay and mortality.
**Holman et al. (** 33 **)**	Population-based cohort study	264,390 T1D	46.6	149,680 (56.6)	464 deaths in T1D had COVID-19 (Feb 16 - May 11, 2020); 1604 deaths in T1D (Feb 16-May 11, 2020)	Increase in death of T1D during the pandemic
**Barron et al. (** 34 **)**	Whole-population study	263,830 T1D	46.6	149,330 (56.6)		OR for COVID-19-related death was 3.51 (95% CI 3.16-3.90) for T1D.
**Unsworth et al. (** 35 **)**	Multicenter Regional Findings	33 patients	10.9	22 (68)	12-15 more NT1D during pandemic	Increase in NT1D during the pandemic Increase in DKA incidence and severity during the pandemic
**Cariou et al (** 19 **)**	Multicenter observational study, diabetic patients hospitalized for COVID-19	1317	69.8	855 (64.9)	10.6% death; 41 (3.1%) NT1D	No increased death in T1D. No death in T1D patients younger than 65 years
		1166 T2D (88.5%)			1.00 OR for death; 41 (3.1%) NT1D	
		39 T1D (3%)			0.44 OR for death; 41 (3.1%) NT1D	
**Mariet et al. (** 31 **)**	Nationwide retrospective cohort study in three periods: week 2 of 2019 to week 12 of 2020, weeks 12-19 of 2020, week 19 of 2020 to week 52 of 2021 (after lockdown)	7,995,449	1 - 35		T1D hospitalizations: 6114 in 2019; 6051 in 2020; 6593 in 2021	No significant increase in the hospitalizations rate for NT1D during the COVID-19 pandemic within 2020 and 2021. The severity of T1D at diagnosis was not exaggerated during the COVID-19 pandemic.
**Kamrath et al. (** 10 **)**	Multicenter cohort study, German Diabetes Registry, NT1D within March 1 and June 30	1,072 in 2020	10.0	430 (58.7)	6.6% (5.1-8.4); NT1D 7.2% (6.5-8.0) NT1D	No significant increase in new-onset autoantibody-negative T1D in children, adolescents, and young adults during the pandemic. No increased susceptibility to DKA in autoantibody-negative T1D before or during the pandemic
		8,349 (2011-2019)	10.1	3033 (53.9)		
**Kamrath et al. (** 11 **)**	A multicenter cohort from the German Diabetes Prospective Follow-up Registry	3238 NT1D in 2020	9.8	1799 (55.6)	DKA cases 1094 (33.8%). An increase in the incidence of COVID-19 or death was associated with DKA RR of 1.40 (95% CI: 1.10-1.77; P = 0.006) and 1.23 (1.14-1.32; P < 0.001), respectively.	Significant increase in the risks of DKA and severe DKA in children with NT1D during the coronavirus pandemic in Germany. Ketoacidosis incidence in 2020 ranged from 22.6% in January to 43.3% in August (expected 20.1% in January to 25.3% in October). Ketoacidosis observed in 2020 in children with NT1D vs. expected rates (2000 to 2019)
**Kamrath et al. (** 20 **)**	Multicenter Diabetes Prospective Study, German Registry, T1D incidence in children and adolescents 1/1/2020 -6/30/2021	5,162 within 2020/2021	9.7	(55.8 )	24.4% (23.6-25.2) NT1D incidence 2020/21 21.2% (20.5-21.9). NT1D expected incidence within 2011 to 2019; IRR 1.15 (1.10-1.20); P < 0.001	IRR 1.15 (95% CI: 1.10-1.20; P < 0.001). IRR in females, 1.14 (95% CI: 1.07-1.21, P < 0.001) and males, 1.16 (95% CI: 1.10-1.23, P < 0.001). Significant increase in IRR in children aged < 6 years (IRR = 1.23, 95% CI: 1.13-1.33, P < 0.001) and 6-11 years (IRR = 1.18, 95% CI: 1.11-1.26, P < 0.001), but not in adolescents aged 12-17 years (IRR = 1.06, 95% CI: 0.98-1.13, P = 0.13)
		2,740 in 2018	9.8	(55.0 )		
		2,903 in 2019	9.7	(54.9 )		
**Jacob et al. (** 21 **)**	A retrospective cross-sectional study, 11 Israeli pediatric Eds, diabetes-related presentation	150 T1D; 48,176 visits (2020)	12		DKA in established T1D 2020 vs. 2019 (59.3% vs. 41.9%, P < 0.043). DKA in NT1D 2020 vs. 2019 (53.4% vs. 38.7%, P = 0.063). No significant increase in NT1D	Significant increase in DKA rate in established T1D. Non-statistically significant increase in DKA rate in NT1D. No difference in severe DKA (established T1D [15.6% vs. 8.1%; P = 0.184] and newly diagnosed T1D [18.6% vs. 17.5%; P = 0.858])
		154 T1D 77,477 visits (2019)	12			
**Mastromauro et al. (** 12 **)**	Retrospective, Pediatric and Adolescent T1D Group 1/2015 - 2/2020 Group 2 3/2020 - 4/2021	172 NT1D; 132 group 1; 40 group 2	9.1; 9.3; 8.4	101 (58.7); 81 (61.3); 420 (50)	DKA (36% vs. 55%, P = 0.03); Severe DKA (8.4% vs. 22.5%, P = 0.01)	Significant increase in DKA and severe DKA during the pandemic
**Dzygalo et al. (** 22 **)**	Observational retrospective cohort study, children 0-18 years with newly diagnosed T1D	34 group 2020; 52 group 2019; (March-May)	9.90; 9.59	22 (64.7); 26 (50)	DKA (52.94% vs. 40.38%, P = 0.276); Severe DKA (32.35% vs. 11.54%, P = 0.0262)	The DKA rate has increased by 12%. Severe DKA cases noted in newly diagnosed T1D children
**Ho et al. (** 23 **)**	Retrospective study, < 18 years, NT1D during the pandemic; March 17 to August 31, 2020 vs. 2019	107 NT1D in 2020	9.62	46 (43.0)	No significant increase in NT1D; Higher DKA (68.2% vs. 45.6%; P < 0.001) and higher severe DKA (27.1% vs. 13.2%; P = 0.01) in 2020 vs. 2019	Significant increase in DKA and severe DKA in NT1D children during the COVID-19 pandemic period
		114 NT1D in 2019	9.43	47 (41.2)		
**Zubkiewicz Kucharska et al. (** 13 **)**	Multicenter cohort study, the T1D pediatric registry for Lower Silesia (children aged 0-18 years) IR within 2000-2019 vs. first 4 months in 2020		0-18		36.67% DKA incidence in 2020 vs. 31.75% DKA incidence within 2000-2019 (P > 0.05); T1D cases (March, April) 2020 were half of the same months in 2019 (P > 0.05). IRT1D 17.27/100,000/year in 2020 vs. IRT1D 17.51/100,000/year within 2000-2019. IRT1D in 2020 (first 4 months) was significantly lower than the period 201-2019 (P = 0.0016), but comparable to 2019 (P = 0.0808)	Increase in IR of T1D 2000 -2019: 10.43/100,000/year in 2000; 22.06/100,000/year in 2019; 27.10/100,000/year, peak incidence in 2017 Highest T1D incidence rate in January and February; DKA incidence: 23.65% within 2000-2004; 34.23% within 2005-2009; 35.59% within 2010-2014; 36.71% within 2015-2019. The IR of T1D during the COVID-19 pandemic was comparable, although their clinical condition was worse.
		1961 within 2000-2019	0-18	1054 (53.72)		
**Pietrzak et al. (** 24 **)**	Multicenter cohort study, DKA incidence in T1D COVID-19 (15/3/2020-15/3/2021) and before COVID-19 (15/2/2019-15/3/2020)	3062 T1D; 1347 (44%) DKA	9.5	1632 (53.3)	826 (49.4%) within 2020/2021; IR 25.90 cases/100000; 1671 (54.6%) within 2020/2021	COVID-19 was associated with an increase in the frequency of DKA and its severity.
					521 (37.5%) within 2019/2020; IR 21.55 cases/100,000; 1391 (45.4%) within 2019/2020	
**Boboc et al. (** 25 **)**	Observational retrospective cohort study, pediatric T1D patient from Marie Curie Emergency Children’s Hospital, Bucharest.	147 (3/2020-2/2021)	7.59	243 (53)	65.99% DKA; 13.2 NT1D/month (5/2020-2/2021)	An increase in the incidence and severity of T1D in children during the COVID-19 pandemic; 30.08% increase in NT1D during the pandemic; 67.40% increase in DKA incidence during the pandemic
		312 (2003-2019)			39.42% DKA; 9.4 NT1D/month (5/2018-2/2019)	
**Alaqeel et al. (** 26 **)**	Multicenter retrospective cohort study, 1-14 years admitted with NT1D or DKA during the COVID-19 pandemic	106 (March-June 2020)	10	51 (48.1)	NT1D 41 (38.7%); DKA 88 (83%); DKA frequency NT1D 23 (26%)	DKA was higher in 2020 vs. 2019 (83% vs. 73%; P = 0.05; risk ratio = 1.15; 95% CI: 1.04-1.26). DKA frequency among NT1D was higher in 2020 vs. 2019 (26% vs. 13.4%; P ≤ 0.001)
		154 (March-June 2019)	9.7	69 (44.8)	NT1D 57 (37.0%); DKA 112 (72.7%); DKA frequency NT1D 15 (13.4%)	
**Dilek et al. (** 27 **)**	Cross-sectional study, newly diagnosed with T1D in Cukurova University hospital	74 (2020)	10	35 (47.3)	DKA 68 (91.9%); Moderate DKA 16 (23.5%) Severe DKA 15 (22.1%)	Increase in the number of NT1D, autoantibody positivity, rates, and severity of DKA during the COVID-19 pandemic
		46 (2019)	10.5	21 (45.7)	DKA 27 (58.7%); Moderate DKA 5 (18.5%); Severe DKA 4 (14.8%)	
**O’Malley et al. (** 28 **)**	Multicenter cross-sectional, adults over the age of 19 years with T1D and COVID-19	113 (March 1, 2020 - August 22, 2020)	39.9	55 (48.7)	Death 5 (4.4%); DKA 27 (23.8%)	TID is associated with a higher risk of morbidity and mortality in COVID-19 patients.
**Bogale et al. (** 29 **)**	Retrospective analysis, all pediatric patients (age ≤ 18 years) newly diagnosed with T1D (01/01/2017 - 09/14/2020)	42 post-COVID	9.2	23 (54.8%)	DKA 20 (47.6%); Moderate or severe DKA 13 (31.0%)	Almost similar DKA rates and severity during COVID-19
		370 pre-COVID	10	218 (58.9%)		
**Danne et al. (** 30 **)**	Retrospective cohort, T1D ≤ 21 years of age, 22,820 May/June 21,820; August/September 2019 and 2020	12,157 (M/J2020)	13.5	52%	T1D duration 4.5; At least one DKA 1.1%; At least 1 severe hypo 0.3%	A significant rise in DKA rate and mortality during COVID-19
		13,386 (A/S 2020)	13.6	51.9%	T1D duration 4.6; At least 1 DKA 0.7%; At least 1 severe hypo 0.3%	
		16,735 (M/J 2019)	13.4	51.7%	T1D duration 4.5; At least 1 DKA 0.8%; At least 1 severe hypo 0.5%	
		14,523 (A/S 2019	13.4	51.6%	T1D duration 4.6; At least 1 DKA 1.0%; At least 1 severe hypo 0.5%	
**Trieu et al. (** 32 **)**	Hospitalized children with T1D or T2D and SARS-CoV-2 infection within April and November 2020	9 NT1D + COVID	10.5	2 (22%)	DKA 64.3% in 2020; DKA 56.9% in 2019; DKA 47.1% in 2018 ; NT1D 286 children in 2020; NT1D 246 children in 2019; NT1D 263 children in 2018	16.3% increased rate of NT1D in 2020; 6.5% decrease in NT1D within 2018 to 2019; Increase in DKA incidence in 2020
		12 known T1D + COVID	12.4	6 (50%)		
**Kendall et al. (** 36 **)**	Global Collaborative Network, 74 large healthcare organizations across 50 US states and 14 countries	1,091,494 pediatric 314,917 COVID-19; 776,577 respiratory infections (not COVID-19)	9.3	143 289 (50.2%)	123 (0.043%) NT1D 6 months after COVID; 72 (0.025%) NT1D 6 months after non-COVID-19 respiratory infection	Risk of NT1D after SARS-CoV-2 infection: 3 months: HR, 2.10 (95% CI: 1.48-3.00) 6 months: HR, 1.83 (95% CI: 1.36-2.44)

Abbreviations: CI, confidence interval; COVID-19, coronavirus disease 2019; DKA, diabetic ketoacidosis; IR, incidence rate; IRR, incidence rate ratio; IRT1D, incidence rate of type 1 diabetes; NT1D, new-onset type 1 diabetes; OR, odds ratio; RR, relative risk; SARS-CoV-2, severe acute respiratory syndrome coronavirus 2; T1D, type 1 diabetes; T2D, type 2 diabetes.

**Table 2.** Summary of Studies’ Outcomes for New-Onset Type 1 Diabetes (NT1D) and Complications During the Coronavirus Disease 2019 (COVID-19) Pandemic, Listing the Countries in Alphabetical Order

Country	NT1D	Complications (e.g., Death and DKA)
**International multicenter study (** 7 **)**		Increased DKA prevalence
**Australia (** 8 **)**	No increase in NT1D	Increase in severe DKA
**China (** 9 **)**		Increased DKA and severity
**England (**33-35**)**	Increased NT1D	Increased DKA and severity; Increase in death in T1D
**French (** 19 **, ** 31 **)**		No increased death in T1D; No significant increased hospitalizations; No increased severity of T1D at diagnosis
**Germany (** 10 **, ** 11 **, ** 20 **)**	Increased NT1D No significant increase in NT1D autoantibody-negative	Increased DKA and severity; No increased DKA in autoantibody-negative T1D
**Hungary (** 37 **)**	Increased NT1D	
**Israel (** 21 **)**	No significant increase in NT1D	Increased DKA significantly but not severe DKA; Increased DKA in NT1D but not significant
**Italy (** 12 **)**		Increased DKA and severity
**Poland (**13**, **22-24**)**	No significant increase in NT1D	Increased DKA and severity
**Romania (** 25 **)**	Increased NT1D	Increased DKA
**Saudi Arabia (** 26 **)**	No increase in NT1D	Increased DKA
**Turkey (** 27 **, ** 38 **)**	Increased NT1D and autoantibody positivity No clear association between SARS-CoV-2 infection and NT1D	Increased DKA and severity
**USA (**28-30**, **32**, **36**)**	Increased NT1D	Increased or similar DKA; Increased mortality and morbidity

Abbreviations: NT1D, new-onset type 1 diabetes; DKA, diabetic ketoacidosis; SARS-CoV-2, severe acute respiratory syndrome coronavirus 2; T1D, type 1 diabetes.

Study Characteristics. Among the 25 eligible studies, there were 15 (60%) multicenter or nationwide studies (7, 10, 11, 13, 19, 20, 24, 26, 28, 30, 31, 33-36). New-onset type 1 diabetes incidence was reported in 13 manuscripts (52%) (8, 10, 13, 20, 21, 23-27, 32, 35, 36) from countries other than Hungary (37) and Turkey (38), which are not included in Table 1. Eighteen studies reported DKA incidence or severity (7-13, 21-27, 29, 30, 32, 35).

COVID-19 and T1D Incidence. The incidence of NT1D in children was increased in England (35), Germany (20), Hungary (37), Romania (25), Turkey (27), and USA (32, 36) but not in Australia (8), Israel (21), Poland (13, 23, 24), Saudi Arabia (26), and Turkey (38) during the COVID-19 pandemic. Furthermore, there was no significant increase in new-onset autoantibody-negative T1D in children, adolescents, and young adults in Germany (10); nevertheless, NT1D and autoantibody positivity were higher in Turkey (27) during the pandemic.

Clinical Outcomes and Complications of T1D Patients During COVID-19. Diabetic ketoacidosis, hospitalization rate, and death were studied in the selected studies. Diabetic ketoacidosis incidence, prevalence, or severity were increased in most studies (7-9, 11-13, 21-27, 30, 32) during the COVID-19 pandemic. However, there was no increase in DKA incidence in autoantibody-negative T1D in Germany (10) or in NT1D in Israel (21). There was also no increase in severe DKA in Israel (21) and no increase in DKA or severity in the USA (29).

The hospitalization rate of NT1D during the pandemic appeared to be stable in France (31). However, the data regarding the death rate is inconsistent, as increases in death were reported in China (9), England (33, 34), and the USA (28, 30); however, no increase in death was observed in France (19).

## 4. Discussion

In this narrative review, most studies reported an increase in DKA incidence and severity during the COVID-19 pandemic. Diabetic ketoacidosis is a serious complication of diabetes, which is associated with more severe pancreatic β-cell destruction and increased morbidity and mortality (39). There is generally a slight increase in the prevalence of DKA at the onset of T1D, which is estimated to be around 29.9%. However, the prevalence of DKA varies across the countries with the lowest prevalence, namely Sweden (19.5%) and Denmark (20.7%), and the highest prevalence, namely Luxembourg (43.8%) and Italy (41.2%) (40). Furthermore, the overall incidence of DKA is declining in Denmark (41) but increases through adolescence in England and Wales (42). Therefore, the incidence and severity of DKA could be additionally affected by COVID-19, which resulted in a relatively consistent increase in DKA incidence and severity across countries (Table 2) during the COVID-19 pandemic. This potentially underscores the underlying COVID-19-related mechanisms, including multisystem inflammatory response (43) and the exacerbation of insulin resistance (44). Additionally, the delayed diagnosis and heterogeneous presentation of NT1D during the COVID-19 pandemic could be further contributing factors (45).

Severe acute respiratory syndrome coronavirus 2 enters human cells mainly through the angiotensin-converting enzyme 2 (ACE2) receptor. There are other receptors that might mediate SARS-CoV-2 entry into human cells, including dipeptidyl peptidase 4 (DPP-4 or CD26), CD147, neuropilin-1, lectins, CD209L, and tyrosine-protein kinase receptor UFO (AXL). The host proteases, such as transmembrane protease serine 2 (TMPRSS2), furin, trypsin, elastase, and cathepsin L, are also involved in the process of SARS-CoV-2 entry into cells. Angiotensin-converting enzyme 2 on cell membrane has other responsibilities against inflammation, proliferation, and fibrosis.

**Figure 2.**
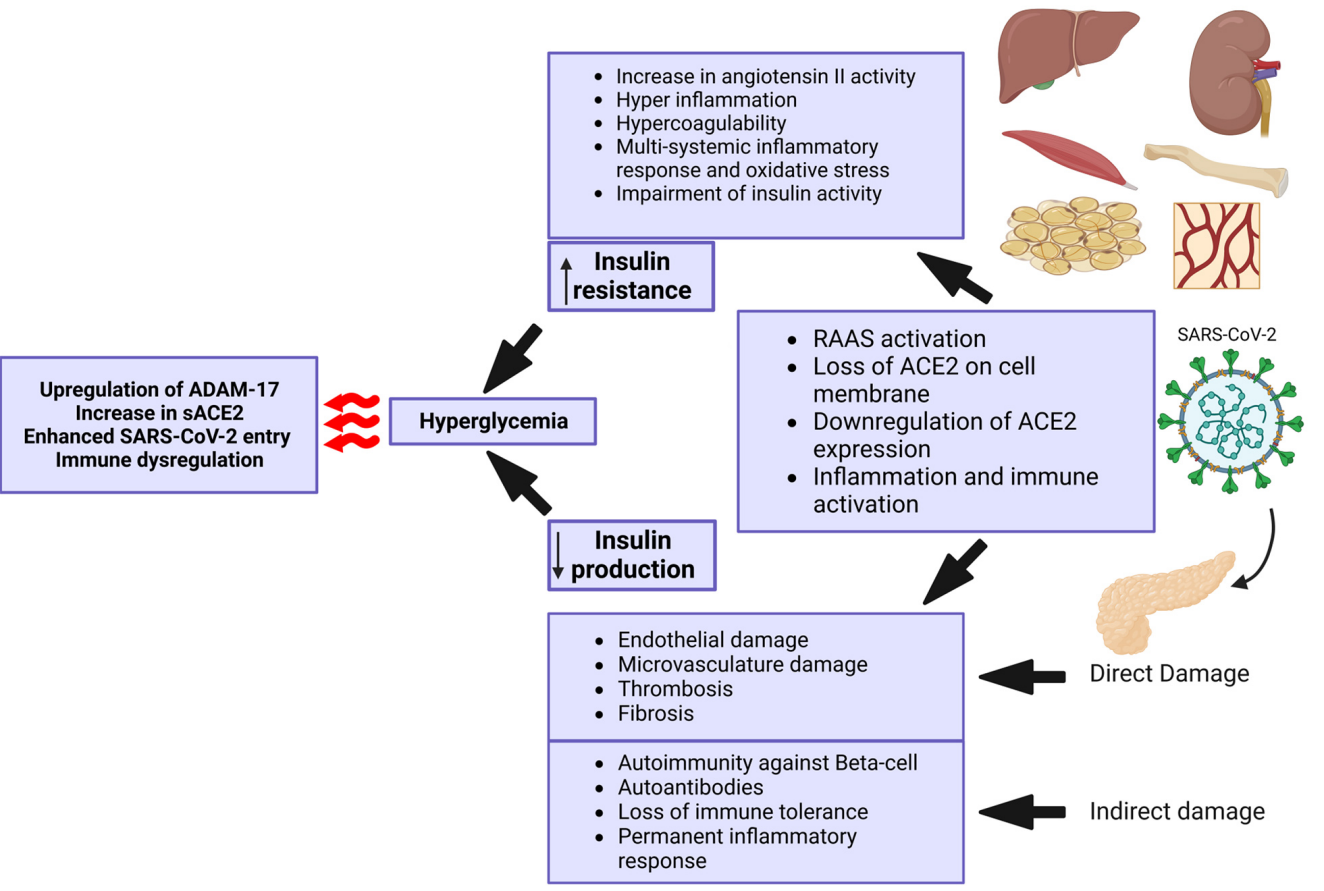
Illustrating the cascade of events triggered by severe acute respiratory syndrome coronavirus 2 (SARS-CoV-2) infection, which results in reduced insulin production, increased insulin resistance, and hyperglycemia. Created with BioRender.com.

The disintegrin and metalloproteinase domain-containing protein 17 (ADAM17) are indirectly involved in the process of SARS-CoV-2 entrance and tissue damage by shedding ACE2 from the cell membrane (46-48). Angiotensin-converting enzyme 2 expression in the gastrointestinal (GI) tract and pancreas is relatively remarkable. It is also expressed in essential metabolic tissues, such as the liver, kidney, adipocytes, and vasculature (49). Coronavirus can potentially target the metabolic tissues, especially the pancreas, which leads to islet cell damage (50), insulin resistance (51), and hyperglycemia (Figure 2). 

Severe acute respiratory syndrome coronavirus 2 can directly infect beta-cells and reduce beta-cell function. It can also lead to beta-cell damage by infecting the surrounding cells, inducing inflammation, and compromising pancreatic blood vessels. In addition, SARS-CoV-2 can trigger autoimmunity that leads to autoimmune pancreatitis and impairment of beta-cell function. Moreover, SARS-CoV-2 infects and replicates in other tissues, such as the kidney, adipose tissue, liver, muscle, bone, and vessels, which causes inflammatory responses, cytokine storms, and multiorgan dysfunction. This, in turn, is associated with insulin resistance, dysglycemia, and even stress hyperglycemia with severe illness. The consequences of these events will increase SARS-CoV-2 infectivity and the risk of further organ damage.

The current information regarding the incidence of NT1D in children during the pandemic is not consistent across countries. This could be due to differences in the outcomes of treatment modalities, accessibility to effective treatment, and speed of conducting a successful approach. However, COVID-19 was associated with the increased incidence and severity of DKA in different countries. Furthermore, there are few case reports or case series, including euglycemic DKA (52), known T1D and DKA (53), or NT1D with or without DKA (43, 54-61) following COVID-19 infection or vaccination (62, 63), which raises concern over not only a possible causal relationship between COVID-19 and T1D but also the beneficial roles of early and practical treatment. In addition, it should be emphasized that the occurrence of new SARS-CoV-2 strains with unique mutations can potentially render resistance to the current antivirals that are routinely used, such as nirmatrelvir/ritonavir, remdesivir, and molnupiravir (64, 65).

Therefore, considering other medications with the capability of targeting the SARS-CoV-2 receptor, reducing virus entry into the cells, and alleviating inflammation might improve clinical outcomes better than antivirals. They might also assist in reducing the risk of hyperglycemia and new-onset diabetes. Ursodeoxycholic acid (UDCA) reduces Farnesoid X receptor (FXR) signaling, downregulates ACE2 expression in the respiratory tract, and diminishes susceptibility to SARS-CoV-2 infection. It was associated with reduced hospitalization, intensive care unit (ICU) admission, and death of COVID-19 patients (66). Ursodeoxycholic acid was also shown to reduce COVID-19 infectivity and severity in cirrhotic patients (67).

Antiandrogens downregulate TMPRSS2 and ACE2, which reduce SARS-CoV-2 entry into the cells (68). They lower the mortality, hospitalization rate, and duration of SARS-CoV-2 infection (69). Spironolactone, an aldosterone receptor antagonist with anti-androgenic effects, antagonizes TMPRSS2 and ADAM17, reduces virus entry into the cells, and diminishes SARS-CoV-2-mediated endothelial damage (70, 71). It has been reported that spironolactone improves clinical scores and reduces mortality, ICU admission, intubation, and end-organ damage in hospitalized COVID-19 patients (71).

Metformin activates AMP-activated protein kinase (AMPK), which leads to the phosphorylation of ACE2. Angiotensin-converting enzyme 2 phosphorylation enhances ACE2 stability on the cell membrane, increases Ang (angiotensin) 1-7 and endothelial nitric oxide synthase bioavailability, and thereby provides lung protection by preserving endothelial function. Additionally, the phosphorylation of ACE2 might affect virus entry into the cells. Metformin also inhibits the mammalian target of the rapamycin (mTOR) pathway and modulates the immune response against the infection (72-74). Generally, metformin seems to be helpful in reducing SARS-CoV-2-related tissue injury. Metformin could not improve the clinical outcomes of COVID-19 patients impressively (75); however, it could reduce the incidence of long COVID (76). Dipeptidyl peptidase 4 inhibitors have immunomodulatory roles and possibly blunt the alternative route of virus entry through DPP-4 receptors (47). They can alleviate SARS-CoV-2 cytokine storm and injury to the organs. The use of DPP-4 inhibitors in patients with SARS-CoV-2 infection was associated with the improvement of glucose levels in diabetic patients and clinical improvement and reduction of inflammatory markers in diabetic and non-diabetic patients (71, 77, 78).

There are some limitations in the current systematic review. This study did not review all resources and did not have information from all areas of the world, which technically limits the applicability of the results for the missing regions of the world. In addition, the time of data collection during the pandemic is not similar in all studies, and it is possible that the incidence of T1D was transiently affected during the pandemic. However, in a meta-analysis by D’Souza et al. (17), there was a higher incidence rate of T1D during the first year (incidence rate ratio [IRR] = 1.14; 95% CI: 1.08 - 1.21) and second year (IRR = 1.27; 95% CI: 1.18 - 1.37) of the pandemic than the period before the pandemic.

### 4.1. Conclusions

Based on the collected evidence, the effect of SARS-CoV-2 infection on the incidence of NT1D is controversial. However, COVID-19 increases the incidence and severity of DKA in T1D patients. Antivirals seem to be helpful but not completely protective against SARS-CoV-2-induced tissue injuries. An alternative therapeutic approach includes targeting the SARS-CoV-2 receptor, blocking virus entry, and alleviating inflammation, especially by combining medications with different beneficial characteristics, to tackle SARS-CoV-2 infection and associated complications. Flooring the path for future clinical trials to investigate the protective role of this alternative approach would be reasonable, as it is shown that the combination of spironolactone and sitagliptin could reduce the hospitalization rate and duration of the disease (79).

## References

[1] Rabaan AA, Smajlovic S, Tombuloglu H, Cordic S, Hajdarevic A, Kudic N (2023). SARS-CoV-2 infection and multi-organ system damage: A review.. Biomol Biomed..

[2] Clarke SA, Abbara A, Dhillo WS (2022). Impact of COVID-19 on the Endocrine System: A Mini-review.. Endocrinology..

[3] Banday MZ, Sameer AS, Nissar S (2020). Pathophysiology of diabetes: An overview.. Avicenna J Med..

[4] Kim SH, Arora I, Hsia DS, Knowler WC, LeBlanc E, Mylonakis E (2023). New-Onset Diabetes After COVID-19.. J Clin Endocrinol Metab..

[5] Wang Y, Guo H, Wang G, Zhai J, Du B (2023). COVID-19 as a Trigger for Type 1 Diabetes.. J Clin Endocrinol Metab..

[6] Muniangi-Muhitu H, Akalestou E, Salem V, Misra S, Oliver NS, Rutter GA (2020). Covid-19 and Diabetes: A Complex Bidirectional Relationship.. Front Endocrinol (Lausanne)..

[7] Birkebaek NH, Kamrath C, Grimsmann JM, Aakesson K, Cherubini V, Dovc K (2022). Impact of the COVID-19 pandemic on long-term trends in the prevalence of diabetic ketoacidosis at diagnosis of paediatric type 1 diabetes: an international multicentre study based on data from 13 national diabetes registries.. Lancet Diabetes Endocrinol..

[8] Lawrence C, Seckold R, Smart C, King BR, Howley P, Feltrin R (2021). Increased paediatric presentations of severe diabetic ketoacidosis in an Australian tertiary centre during the COVID-19 pandemic.. Diabet Med..

[9] Li J, Wang X, Chen J, Zuo X, Zhang H, Deng A (2020). COVID-19 infection may cause ketosis and ketoacidosis.. Diabetes Obes Metab..

[10] Kamrath C, Rosenbauer J, Tittel SR, Warncke K, Hirtz R, Denzer C (2021). Frequency of Autoantibody-Negative Type 1 Diabetes in Children, Adolescents, and Young Adults During the First Wave of the COVID-19 Pandemic in Germany.. Diabetes Care..

[11] Kamrath C, Rosenbauer J, Eckert AJ, Pappa A, Reschke F, Rohrer TR (2021). Incidence of COVID-19 and Risk of Diabetic Ketoacidosis in New-Onset Type 1 Diabetes.. Pediatrics..

[12] Mastromauro C, Blasetti A, Primavera M, Ceglie L, Mohn A, Chiarelli F (2022). Peculiar characteristics of new-onset Type 1 Diabetes during COVID-19 pandemic.. Ital J Pediatr..

[13] Zubkiewicz Kucharska A, Seifert M, Stepkowski M, Noczynska A (2021). Diagnosis of type 1 diabetes during the SARS-CoV-2 pandemic: Does lockdown affect the incidence and clinical status of patients?. Adv Clin Exp Med..

[14] Nassar M, Nso N, Baraka B, Alfishawy M, Mohamed M, Nyabera A (2021). The association between COVID-19 and type 1 diabetes mellitus: A systematic review.. Diabetes Metab Syndr..

[15] Zhang T, Mei Q, Zhang Z, Walline JH, Liu Y, Zhu H (2022). Risk for newly diagnosed diabetes after COVID-19: a systematic review and meta-analysis.. BMC Med..

[16] Rahmati M, Keshvari M, Mirnasuri S, Yon DK, Lee SW, Il Shin J (2022). The global impact of COVID-19 pandemic on the incidence of pediatric new-onset type 1 diabetes and ketoacidosis: A systematic review and meta-analysis.. J Med Virol..

[17] D'Souza D, Empringham J, Pechlivanoglou P, Uleryk EM, Cohen E, Shulman R (2023). Incidence of Diabetes in Children and Adolescents During the COVID-19 Pandemic: A Systematic Review and Meta-Analysis.. JAMA Netw Open..

[18] Moher D, Shamseer L, Clarke M, Ghersi D, Liberati A, Petticrew M (2015). Preferred reporting items for systematic review and meta-analysis protocols (PRISMA-P) 2015 statement.. Syst Rev..

[19] Cariou B, Hadjadj S, Wargny M, Pichelin M, Al-Salameh A, Allix I (2020). Phenotypic characteristics and prognosis of inpatients with COVID-19 and diabetes: the CORONADO study.. Diabetologia..

[20] Kamrath C, Rosenbauer J, Eckert AJ, Siedler K, Bartelt H, Klose D (2022). Incidence of Type 1 Diabetes in Children and Adolescents During the COVID-19 Pandemic in Germany: Results From the DPV Registry.. Diabetes Care..

[21] Jacob R, Weiser G, Krupik D, Takagi D, Peled S, Pines N (2021). Diabetic Ketoacidosis at Emergency Department Presentation During the First Months of the SARS-CoV-2 Pandemic in Israel: A Multicenter Cross-Sectional Study.. Diabetes Ther..

[22] Dzygalo K, Nowaczyk J, Szwilling A, Kowalska A (2020). Increased frequency of severe diabetic ketoacidosis at type 1 diabetes onset among children during COVID-19 pandemic lockdown: an observational cohort study.. Pediatr Endocrinol Diabetes Metab..

[23] Ho J, Rosolowsky E, Pacaud D, Huang C, Lemay JA, Brockman N (2021). Diabetic ketoacidosis at type 1 diabetes diagnosis in children during the COVID-19 pandemic.. Pediatr Diabetes..

[24] Pietrzak I, Michalak A, Seget S, Bednarska M, Ben-Skowronek I, Bossowski A (2022). Diabetic ketoacidosis incidence among children with new-onset type 1 diabetes in Poland and its association with COVID-19 outbreak-Two-year cross-sectional national observation by PolPeDiab Study Group.. Pediatr Diabetes..

[25] Boboc AA, Novac CN, Ilie MT, Iesanu MI, Galos F, Balgradean M (2021). The Impact of SARS-CoV-2 Pandemic on the New Cases of T1DM in Children. A Single-Centre Cohort Study.. J Pers Med..

[26] Alaqeel A, Aljuraibah F, Alsuhaibani M, Huneif M, Alsaheel A, Dubayee MA (2021). The Impact of COVID-19 Pandemic Lockdown on the Incidence of New-Onset Type 1 Diabetes and Ketoacidosis Among Saudi Children.. Front Endocrinol (Lausanne)..

[27] Dilek SO, Gurbuz F, Turan I, Celiloglu C, Yuksel B (2021). Changes in the presentation of newly diagnosed type 1 diabetes in children during the COVID-19 pandemic in a tertiary center in Southern Turkey.. J Pediatr Endocrinol Metab..

[28] O'Malley G, Ebekozien O, Desimone M, Pinnaro CT, Roberts A, Polsky S (2021). COVID-19 Hospitalization in Adults with Type 1 Diabetes: Results from the T1D Exchange Multicenter Surveillance Study.. J Clin Endocrinol Metab..

[29] Bogale KT, Urban V, Schaefer E, Bangalore Krishna K (2021). The Impact of COVID-19 Pandemic on Prevalence of Diabetic Ketoacidosis at Diagnosis of Type 1 Diabetes: A Single-Centre Study in Central Pennsylvania.. Endocrinol Diabetes Metab..

[30] Danne T, Lanzinger S, de Bock M, Rhodes ET, Alonso GT, Barat P (2021). A Worldwide Perspective on COVID-19 and Diabetes Management in 22,820 Children from the SWEET Project: Diabetic Ketoacidosis Rates Increase and Glycemic Control Is Maintained.. Diabetes Technol Ther..

[31] Mariet AS, Petit JM, Benzenine E, Quantin C, Bouillet B (2023). Incidence of new-onset type 1 diabetes during Covid-19 pandemic: A French nationwide population-based study.. Diabetes Metab..

[32] Trieu C, Sunil B, Ashraf AP, Cooper J, Yarbrough A, Pinninti S (2021). SARS-CoV-2 infection in hospitalized children with type 1 and type 2 diabetes.. J Clin Transl Endocrinol..

[33] Holman N, Knighton P, Kar P, O'Keefe J, Curley M, Weaver A (2020). Risk factors for COVID-19-related mortality in people with type 1 and type 2 diabetes in England: a population-based cohort study.. Lancet Diabetes Endocrinol..

[34] Barron E, Bakhai C, Kar P, Weaver A, Bradley D, Ismail H (2020). Associations of type 1 and type 2 diabetes with COVID-19-related mortality in England: a whole-population study.. Lancet Diabetes Endocrinol..

[35] Unsworth R, Wallace S, Oliver NS, Yeung S, Kshirsagar A, Naidu H (2020). New-Onset Type 1 Diabetes in Children During COVID-19: Multicenter Regional Findings in the U.K.. Diabetes Care..

[36] Kendall EK, Olaker VR, Kaelber DC, Xu R, Davis PB (2022). Association of SARS-CoV-2 Infection With New-Onset Type 1 Diabetes Among Pediatric Patients From 2020 to 2021.. JAMA Netw Open..

[37] Herczeg V, Luczay A, Tenai N, Czine G, Toth-Heyn P (2022). Anti-SARS-CoV-2 Seropositivity Among Children With Newly Diagnosed Type 1 Diabetes Mellitus: A Case-Control Study.. Indian Pediatr..

[38] Ata A, Jalilova A, Kirkgoz T, Isiklar H, Demir G, Altinok YA (2022). Does COVID-19 predispose patients to type 1 diabetes mellitus?. Clin Pediatr Endocrinol..

[39] Dhatariya KK, Glaser NS, Codner E, Umpierrez GE (2020). Diabetic ketoacidosis.. Nat Rev Dis Primers..

[40] Cherubini V, Grimsmann JM, Akesson K, Birkebaek NH, Cinek O, Dovc K (2020). Temporal trends in diabetic ketoacidosis at diagnosis of paediatric type 1 diabetes between 2006 and 2016: results from 13 countries in three continents.. Diabetologia..

[41] Stougaard EB, Amadid H, Sondergaard E, Carstensen B, Jorgensen ME, Norgaard K (2023). Time Trends in the Incidence of Diabetic Ketoacidosis Leading to Hospital Admission Among Adults With Type 1 Diabetes-A Nationwide Danish Register Study.. Diabetes Care..

[42] Holman N, Woch E, Dayan C, Warner J, Robinson H, Young B (2023). National Trends in Hyperglycemia and Diabetic Ketoacidosis in Children, Adolescents, and Young Adults With Type 1 Diabetes: A Challenge Due to Age or Stage of Development, or Is New Thinking About Service Provision Needed?. Diabetes Care..

[43] Aly HH, Fouda EM, Kotby AA, Magdy SM, Rezk AR, Nasef MWA (2022). COVID-19-Related Multisystem Inflammatory Syndrome in Children Presenting With New-Onset Type 1 Diabetes in Severe Ketoacidosis: A Case Series.. Diabetes Care..

[44] Keiner ES, Slaughter JC, Datye KA, Cherrington AD, Moore DJ, Gregory JM (2022). COVID-19 Exacerbates Insulin Resistance During Diabetic Ketoacidosis in Pediatric Patients With Type 1 Diabetes.. Diabetes Care..

[45] Rabbone I, Schiaffini R, Cherubini V, Maffeis C, Scaramuzza A, Diabetes Study Group of the Italian Society for Pediatric E (2020). Has COVID-19 Delayed the Diagnosis and Worsened the Presentation of Type 1 Diabetes in Children?. Diabetes Care..

[46] Jackson CB, Farzan M, Chen B, Choe H (2022). Mechanisms of SARS-CoV-2 entry into cells.. Nat Rev Mol Cell Biol..

[47] Bakhtiari M, Asadipooya K (2022). Metainflammation in COVID-19.. Endocr Metab Immune Disord Drug Targets..

[48] Brojakowska A, Narula J, Shimony R, Bander J (2020). Clinical Implications of SARS-CoV-2 Interaction With Renin Angiotensin System: JACC Review Topic of the Week.. J Am Coll Cardiol..

[49] Hikmet F, Mear L, Edvinsson A, Micke P, Uhlen M, Lindskog C (2020). The protein expression profile of ACE2 in human tissues.. Mol Syst Biol..

[50] Liu F, Long X, Zhang B, Zhang W, Chen X, Zhang Z (2020). ACE2 Expression in Pancreas May Cause Pancreatic Damage After SARS-CoV-2 Infection.. Clin Gastroenterol Hepatol..

[51] Govender N, Khaliq OP, Moodley J, Naicker T (2021). Insulin resistance in COVID-19 and diabetes.. Prim Care Diabetes..

[52] Oriot P, Hermans MP (2022). Euglycemic diabetic ketoacidosis in a patient with type 1 diabetes and SARS-CoV-2 pneumonia: case-report and review of the literature.. Acta Clin Belg..

[53] Gorthi RS, Kamel G, Dhindsa S, Nayak RP (2021). COVID-19 Presenting With Diabetic Ketoacidosis: A Case Series.. AACE Clin Case Rep..

[54] Benyakhlef S, Abdellaoui W, Tahri A, Rouf S, Latrech H (2021). Diabetic Ketoacidosis at Onset of Pediatric Type-1 Diabetes Triggered by Covid-19: An Original Case Report.. Cureus..

[55] Nielsen-Saines K, Li E, Olivera AM, Martin-Blais R, Bulut Y (2021). Case Report: Insulin-Dependent Diabetes Mellitus and Diabetic Keto-Acidosis in a Child With COVID-19.. Front Pediatr..

[56] Soliman AT, Al-Amri M, Alleethy K, Alaaraj N, Hamed N, De Sanctis V (2020). Newly-onset type 1 diabetes mellitus precipitated by COVID-19 in an 8-month-old infant.. Acta Biomed..

[57] Albuali WH, AlGhamdi NA (2021). Diabetic ketoacidosis precipitated by atypical coronavirus disease in a newly diagnosed diabetic girl.. J Taibah Univ Med Sci..

[58] Parappil P, Ghimire S, Saxena A, Mukherjee S, John BM, Sondhi V (2022). New-onset diabetic ketoacidosis with purpura fulminans in a child with COVID-19-related multisystem inflammatory syndrome.. Infect Dis (Lond)..

[59] Genc S, Evren B, Bozbay A, Aydin ES, Genc O, Sahin I (2021). Could Covid-19 Trigger Type 1 Diabetes? Presentation of Covid-19 Case Presented with Diabetic Ketoacidosis.. Acta Endocrinol (Buchar)..

[60] Halioti A, Kitinou M, Chalioti VM, Chaliotis G (2022). SARS-CoV-2 Unmasks Type 1 Diabetes Mellitus With an Episode of Diabetic Ketoacidosis.. J Med Cases..

[61] Taskaldiran I, Nar A (2023). A Case of New-onset Autoimmune Type-1 Diabetes Mellitus Following COVID-19 Infection.. Endocr Metab Immune Disord Drug Targets..

[62] Ganakumar V, Jethwani P, Roy A, Shukla R, Mittal M, Garg MK (2022). Diabetic ketoacidosis (DKA) in type 1 diabetes mellitus (T1DM) temporally related to COVID-19 vaccination.. Diabetes Metab Syndr..

[63] Lin R, Lin YW, Chen MH (2022). Fulminant Type 1 Diabetes Mellitus after SARS-CoV-2 Vaccination: A Case Report.. Vaccines (Basel)..

[64] Hillary VE, Ceasar SA (2023). An update on COVID-19: SARS-CoV-2 variants, antiviral drugs, and vaccines.. Heliyon..

[65] von Delft A, Hall MD, Kwong AD, Purcell LA, Saikatendu KS, Schmitz U (2023). Accelerating antiviral drug discovery: lessons from COVID-19.. Nat Rev Drug Discov..

[66] Brevini T, Maes M, Webb GJ, John BV, Fuchs CD, Buescher G (2023). FXR inhibition may protect from SARS-CoV-2 infection by reducing ACE2.. Nature..

[67] John BV, Bastaich D, Webb G, Brevini T, Moon A, Ferreira RD (2023). Ursodeoxycholic acid is associated with a reduction in SARS-CoV-2 infection and reduced severity of COVID-19 in patients with cirrhosis.. J Intern Med..

[68] Leach DA, Mohr A, Giotis ES, Cil E, Isac AM, Yates LL (2021). The antiandrogen enzalutamide downregulates TMPRSS2 and reduces cellular entry of SARS-CoV-2 in human lung cells.. Nat Commun..

[69] Cheema HA, Rehman AU, Elrashedy AA, Mohsin A, Shahid A, Ehsan M (2023). Antiandrogens for the treatment of COVID-19 patients: A meta-analysis of randomized controlled trials.. J Med Virol..

[70] Fels B, Acharya S, Vahldieck C, Graf T, Kading N, Rupp J (2022). Mineralocorticoid receptor-antagonism prevents COVID-19-dependent glycocalyx damage.. Pflugers Arch..

[71] Abbasi F, Adatorwovor R, Davarpanah MA, Mansoori Y, Hajiani M, Azodi F (2022). A Randomized Trial of Sitagliptin and Spironolactone With Combination Therapy in Hospitalized Adults With COVID-19.. J Endocr Soc..

[72] Zhang J, Dong J, Martin M, He M, Gongol B, Marin TL (2018). AMP-activated Protein Kinase Phosphorylation of Angiotensin-Converting Enzyme 2 in Endothelium Mitigates Pulmonary Hypertension.. Am J Respir Crit Care Med..

[73] Sharma S, Ray A, Sadasivam B (2020). Metformin in COVID-19: A possible role beyond diabetes.. Diabetes Res Clin Pract..

[74] Malhotra A, Hepokoski M, McCowen KC, Y. J. Shyy J (2020). ACE2, Metformin, and COVID-19.. iScience..

[75] Bramante CT, Huling JD, Tignanelli CJ, Buse JB, Liebovitz DM, Nicklas JM (2022). Randomized Trial of Metformin, Ivermectin, and Fluvoxamine for Covid-19.. N Engl J Med..

[76] Bramante CT, Buse JB, Liebovitz DM, Nicklas JM, Puskarich MA, Cohen K (2023). Outpatient treatment of COVID-19 and incidence of post-COVID-19 condition over 10 months (COVID-OUT): a multicentre, randomised, quadruple-blind, parallel-group, phase 3 trial.. Lancet Infect Dis..

[77] Nag S, Mandal S, Mukherjee O, Mukherjee S, Kundu R (2023). DPP-4 Inhibitors as a savior for COVID-19 patients with diabetes.. Future Virol..

[78] Al-Kuraishy HM, Al-Gareeb AI, Qusty N, Alexiou A, Batiha GE (2022). Impact of Sitagliptin on Non-diabetic Covid-19 Patients.. Curr Mol Pharmacol..

[79] Davarpanah MA, Adatorwovor R, Mansoori Y, Ramsheh FSR, Parsa A, Hajiani M (2023). Combination of spironolactone and sitagliptin improves clinical outcomes of outpatients with COVID-19: a prospective cohort study.. J Endocrinol Invest..

